# Maintaining Effective Beta Cell Function in the Face of Metabolic Syndrome-Associated Glucolipotoxicity—Nutraceutical Options

**DOI:** 10.3390/healthcare10010003

**Published:** 2021-12-21

**Authors:** Mark F. McCarty, James J. DiNicolantonio

**Affiliations:** 1 Catalytic Longevity Foundation, San Diego, CA 92109, USA; 2 Department of Preventive Cardiology, Saint Luke’s Mid America Heart Institute, Kansas City, MO 64111, USA

**Keywords:** type 2 diabetes, glucolipotoxicity, beta cells, GSIS, PDX1, JNK, GLP-1, nutraceuticals, pre-biotics, pro-biotics

## Abstract

In people with metabolic syndrome, episodic exposure of pancreatic beta cells to elevated levels of both glucose and free fatty acids (FFAs)—or glucolipotoxicity—can induce a loss of glucose-stimulated insulin secretion (GSIS). This in turn can lead to a chronic state of glucolipotoxicity and a sustained loss of GSIS, ushering in type 2 diabetes. Loss of GSIS reflects a decline in beta cell glucokinase (GK) expression associated with decreased nuclear levels of the pancreatic and duodenal homeobox 1 (PDX1) factor that drives its transcription, along with that of Glut2 and insulin. Glucolipotoxicity-induced production of reactive oxygen species (ROS), stemming from both mitochondria and the NOX2 isoform of NADPH oxidase, drives an increase in c-Jun N-terminal kinase (JNK) activity that promotes nuclear export of PDX1, and impairs autocrine insulin signaling; the latter effect decreases PDX1 expression at the transcriptional level and up-regulates beta cell apoptosis. Conversely, the incretin hormone glucagon-like peptide-1 (GLP-1) promotes nuclear import of PDX1 via cAMP signaling. Nutraceuticals that quell an increase in beta cell ROS production, that amplify or mimic autocrine insulin signaling, or that boost GLP-1 production, should help to maintain GSIS and suppress beta cell apoptosis in the face of glucolipotoxicity, postponing or preventing onset of type 2 diabetes. Nutraceuticals with potential in this regard include the following: phycocyanobilin—an inhibitor of NOX2; agents promoting mitophagy and mitochondrial biogenesis, such as ferulic acid, lipoic acid, melatonin, berberine, and astaxanthin; myo-inositol and high-dose biotin, which promote phosphatidylinositol 3-kinase (PI3K)/Akt activation; and prebiotics/probiotics capable of boosting GLP-1 secretion. Complex supplements or functional foods providing a selection of these agents might be useful for diabetes prevention.

## 1. Failure of Beta Cell Glucose-Stimulated Insulin Secretion Initiates Onset of Type 2 Diabetes

As metabolic syndrome progresses and the insulin insensitivity of peripheral tissues worsens, glycemic control is maintained for some time owing to a compensatory increase in beta cell insulin secretion. However, the episodic exposure of beta cells to elevated levels of both glucose and free fatty acids (FFAs)—a phenomenon known as “glucolipotoxicity”—may eventually induce a loss of beta cell capacity to respond to an acute increase of plasma glucose with an appropriate compensatory increase in insulin secretion [1,2,3,4]. Baseline secretion of insulin may remain normal or high, but beta cells lose their ability to “sense” an increase in plasma glucose, such that postprandial glucose becomes inordinately high, and postprandial FFAs remain high—an effect that further worsens muscle insulin sensitivity. The resulting exacerbation of beta cell glucolipotoxicity may then progress to the point that fasting levels of glucose remain unduly elevated, at which point the patient is diagnosed with type 2 diabetes. The beta cells then remain locked in a dysfunctional state because the glucose and FFA levels are now chronically high.

The failure of glucose-stimulated insulin secretion (GSIS) that characterizes type 2 diabetes has been traced to a reduced expression of beta cell glucokinase activity [5,6]. Healthy beta cells detect an elevation of plasma glucose by increasing the rate at which they take up glucose and metabolize it via glycolysis. The evolved pyruvate is then oxidized in mitochondria, leading to an increase in the rate of mitochondrial ATP generation. The consequent increase in cytoplasmic ATP/ADP ratio inhibits ATP-sensitive potassium channels in the beta cell plasma membrane, such that plasma membrane potential declines. This in turn opens voltage-sensitive calcium channels, causing an influx of calcium that triggers increased exocytosis of beta cell insulin granules [7,8]. The resulting increase in plasma insulin promotes storage of the elevated plasma glucose, such that postprandial glucose eventually returns to its healthy baseline level.

GSIS is made possible by the fact that, unlike most tissues that rely on hexokinase to initiate glycolysis, glucose phosphorylation in beta cells is mediated largely by glucokinase (GK), which is sometimes referred to as the “glucose sensor” of the beta cell [7]. A similar phenomenon is seen in hepatocytes; in the context of elevated plasma glucose, glucose flux through hepatocyte glucokinase serves as a signal to suppress hepatic gluconeogenesis [9]. As contrasted to hexokinase, GK has a higher Michaelis–Menten constant (Km) for glucose, and is not subject to feedback inhibition by glucose-6-phosphate. For this reason, the rate of flux of glucose through GK is roughly proportionate to plasma glucose level, and hence can serve as a signal in beta cells and hepatocytes when plasma glucose is elevated [10]. The rate of ATP generation in healthy beta cells—which determines the rate of insulin secretion—thus varies directly with plasma glucose, which is a homeostatically appropriate response.

## 2. A Key Role for Loss of PDX1 Activity and Glucokinase Expression Driven by ROS 

This mechanism fails in beta cells experiencing excessive glucolipotoxicity owing to a maladaptive reduction in beta cell GK expression [5,6] (expression of the chief beta cell glucose transporter Glut2 also falls, but this does not appear to be rate limiting for beta cell glycolytic activity). The mechanisms responsible for this decline in GK are now understood at least in part, and this understanding may make it feasible to define measures that could help to sustain beta cell GK expression.

The transcription factor PDX1 is a defining feature of beta cells; this transcription factor promotes expression of the key proteins Glut2, GK, and insulin [11]. The nuclear level of PDX1 in beta cells undergoing failure of GSIS notably declines, and this decline can largely account for the concomitant reduction in GK expression. Nuclear PDX1 declines both because of a reduction of its expression at the transcriptional level, and because PDX1 is exported from the nucleus [12,13,14].

The nuclear export of PDX1 in failing beta cells results from its phosphorylation by c-Jun N-terminal kinase (JNK); this activates a nuclear export signal in PDX1 [12]. The elevation of JNK activity that mediates this is driven by increased levels of oxidants, notably hydrogen peroxide [12]. The increase of reactive oxygen species (ROS) in beta cells exposed to glucolipotoxicity appears to reflect increased ROS production by both NADPH oxidase 2 (NOX2) and by mitochondria; the mechanisms driving this increase are still only partially understood, although the increase in mitochondrial oxidant production is at least partially a function of increased Krebs cycle activity, stemming from increased metabolism of glucose and of FFAs [15,16,17,18,19,20]. How ROS activate JNK in failing beta cells also requires further clarification; oxidant-induced ER stress, as well as activation of apoptosis signal-related kinase-1 (ASK-1) may play a role [21,22,23,24]. In any case, measures that suppress the activity of NOX2, and that optimize the structural and functional integrity of mitochondria, such that they can oxidize large amounts of substrate while keeping ROS production at a moderate level, have potential for lessening the JNK-driven nuclear export of PDX1.

## 3. Failure of Autocrine Insulin Signaling Promotes Apoptosis and Reduces PDX1 Expression

The reduction of PDX1 expression induced by glucolipotoxicity may stem largely from a reduction of autocrine insulin signaling within beta cells. Beta cells express insulin receptors, and the insulin-mediated activation of the insulin receptor substrate-2 (IRS-2)/PI3K/Akt pathway both opposes beta cell apoptosis and also supports homeostatically appropriate increases in beta cells’ mass in the context of peripheral insulin sensitivity [25,26,27,28,29]. In beta cells exposed to glucolipotoxicity, insulin-mediated activation of Akt is suppressed. This effect likewise is induced by beta cell oxidant stress; likely, this reflects JNK-mediated phosphorylation of IRS-2, which is known to disrupt its interaction with the insulin receptor and promote its proteolytic degradation [30,31].

The relationship of this phenomenon to PDX1 expression is as follows: appropriate Akt activity phosphorylates the forkhead box O1 (FOXO1) transcription factor, inducing its export from the nucleus [32]. On the promoter of PDX1 gene, FOXO1 competes with the binding of the transcription factor Foxa2, which promotes transcription of this gene [33]. Hence, nuclear FOXO1 acts as a transcriptional repressor of PDX1. Appropriate Akt activity, by inducing the expulsion of FOXO1 from the nucleus, disinhibits transcription of the PDX1 gene, and hence sustains PDX1 expression. The suppression of insulin signaling that characterizes glucolipotoxicity not only exposes beta cells to increased risk of apoptosis—ultimately leading to a maladaptive decrease in beta cell mass—but also compromises PDX1 expression.

In summary, the failure of GSIS induced by glucolipotoxicity, as well as the increased propensity for beta cell apoptosis, appears to reflect the interaction of increased ROS levels and the associated reduction in the efficiency of autocrine insulin signaling. On the positive side, the incretin hormone glucagon-like peptide-1 (GLP-1) acts to support effective GSIS [34]. GLP-1, via stimulation of cAMP production and consequent PKA activation, promotes the nuclear import of PDX1, counteracting the adverse effect of JNK in this regard [35]. Moreover, cAMP-stimulated EPAC (exchange protein activated by cAMP) somehow promotes PDX1 expression [36].

These considerations suggest that nutraceutical or dietary measures that lessen oxidant production by NOX2 or mitochondria, that suppress redox signaling, that reinforce signaling from the insulin receptor to Akt, or that boost intestinal production of GLP-1, could be expected to aid maintenance of GSIS and prevent apoptosis in beta cells challenged by glucolipotoxicity. Such measures may be doubly beneficial if they also counter the peripheral insulin resistance that gives rise to glucolipotoxicity.

## 4. Controlling Beta Cell Oxidant Stress—Focus on NOX2, Mitophagy, and Mitochondrial Biogenesis

With respect to NOX2-mediated oxidant production, the intracellular free bilirubin derived from heme oxygenase activity has been shown to function as a physiological inhibitor of NOX2 activity [37,38,39,40]. This likely explains why, in some prospective epidemiological studies and in Mendelian randomization analysis, higher serum levels of free bilirubin correlate with reduced risk for type 2 diabetes, and why oral administration of biliverdin (rapidly converted to bilirubin within cells) has been found to prevent or postpone the onset of hyperglycemia in db/db mice prone to this diabetes [41,42,43,44]. In this latter study, biliverdin treatment was associated with maintenance of PDX1 and insulin expression in beta cells, and a decrease in beta cell apoptosis, despite the fact that peripheral insulin sensitivity was only modestly influenced. Hence, the data suggested that biliverdin was directly protective to beta cells. Whereas biliverdin is expensive to synthesize and therefore impractical for widescale use as a nutraceutical, its metabolite phycocyanobilin (PCB), a light-absorbing chromophore present in high concentrations in cyanobacteria (such as the food spirulina) and certain blue-green algae, shares the ability of bilirubin to inhibit NOX2, likely because it is converted within cells to phycocyanorubin, a compound that is highly similar to bilirubin [45,46]. This may explain much of the versatile antioxidant and anti-inflammatory activity of spirulina or its chief protein phycocyanin (to which PCB is covalently attached) in a wide range of rodent studies [47,48]. Hence, regular ingestion of adequate amounts of spirulina, or of spirulina extracts enriched in PCB, may have potential for aiding the preservation of proper beta cell function and mass in patients with metabolic syndrome.

As noted, the increased mitochondrial oxidation of substrate that accompanies increased exposure to glucose and FFAs would be expected to increase generation of superoxide by the mitochondrial respiratory chain. However, the extent to which this gives rise to cellular oxidant stress should be modulated by the extent to which mitochondria are structurally and functionally intact. Oxidant-mediated damage to the mitochondrial respiratory chain, or improper formation of this chain owing to oxidant-mediated damage to mitochondrial DNA, could be expected to amplify the rate at which mitochondria release hydrogen peroxide in response to substrate overload. Conversely, increased expression of mitochondrial peroxidase activity would be expected to quell such release.

The process of mitophagy, coupled appropriately to mitochondrial biogenesis (MB), is the mechanism whereby structurally and functionally damaged mitochondria—as detected by a reduction in mitochondrial membrane potential—are replaced by new mitochondria that are more functionally competent and less likely to generate oxidants [49,50]. A previous discussion has proposed that drugs or nutraceuticals that activate sirtuin 1 (Sirt1), AMP-activated protein kinase (AMPK), Nrf2, and peroxisome proliferator-activated receptor alpha (PPARα) could be expected to collaborate in promotion of both mitophagy and MB [51]. Physiologically active nutraceuticals that can boost Sirt1 activity or expression include ferulic acid melatonin [52,53,54,55,56,57,58,59]. The herbal compound berberine, a nutraceutical long employed in diabetes treatment in China, shares the ability of metformin to activate AMPK [60,61,62,63,64]. Nutraceuticals that can amplify the transcriptional activity or expression of Nrf2 include lipoic acid, the sulforaphane generated from broccoli sprout extract, the neurohormone melatonin, and the xanthophyll carotenoid astaxanthin [58,65,66,67,68]. Moreover, astaxanthin can also serve as an agonist for PPARα and can act as a highly effective scavenging antioxidant in the mitochondrial inner membrane [68,69].

Activation of Nrf2 not only aids MB, but also increases mitochondrial expression of peroxidases and superoxide dismutase, which suppress mitochondrial release of hydrogen peroxide and, like astaxanthin, can protect the respiratory chain from oxidative damage [70]. Hence, nutraceuticals that boost Nrf2 activity can do double duty in protecting beta cells from the mitochondrial oxidant stress imposed by glucolipotoxicity. Additionally, by boosting the cytoplasmic expression of various antioxidant enzymes as well as glutathione, they can lessen the ability of hydrogen peroxide to provoke JNK activation [71]. With respect to glutathione, its cellular levels can be further boosted with supplemental N-acetylcysteine (NAC), particularly in the elderly [72,73,74]. In summary, nutraceutical programs featuring some or all of PCB, ferulic acid, melatonin, berberine, lipoic acid, NAC, and astaxanthin may have potential for lessening the activation of JNK induced by glucolipotoxicity. In this regard, each of these agents, excepting PCB, has been shown to protect beta cells from glucolipotoxicity in rodent or cells culture models [75,76,77,78,79,80,81,82,83,84,85,86,87].

Sirt1, in addition to promoting mitophagy and MB, can lessen glucotoxicity-induced mitochondrial oxidant production by preventing JNK-mediated phosphorylation and activation of p66Shc; activated p66Shc can migrate to mitochondria, where it oxidizes cytochrome c to generate hydrogen peroxide [88,89,90]. However, the interaction of JNK with p66Shc requires acetylation of the latter, which Sirt1 can reverse [88].

Support of Sirt1 activity in beta cells stressed by glucolipotoxicity may be all the more important in light of evidence that hyperglycemia can decrease Sirt1 protein expression in these cells [88]. JNK1 activated by glucolipotoxicity can confer a phosphorylation on Sirt1 (at Ser47) that marks it for ubiquitination and subsequent proteasomal degradation [91]. Moreover, recent studies show that USP22 deubiquitinase activity can raise protein levels of Sirt1 by reversing this ubiquitination [92,93,94,95]. The proximal promoter of the USP22 gene contains an Sp1-binding site, and Sp1 binding suppresses the gene’s transcription [96,97]. Activated p38 MAP kinase can confer a phosphorylation on Sp1 that expedites its binding to its response elements and has been found to inhibit Sp1 expression at the transcriptional level [97,98]. Both oxidative and ER stress can drive the activation of p38, in part via apoptosis signaling-regulated kinase (ASK1) [71,99,100]. Hence, oxidative and ER stress have the potential to decrease the protein expression of Sirt1 by inhibiting expression of USP22 deubiquitinase [90,97]. Indeed, in cell lines derived from human pancreatic beta cells or an insulinoma, hyperglycemia-induced NADPH oxidase activity, via p38, has been found to suppress transcription of the USP22 gene, and thereby down-regulate protein expression of Sirt1 [88].

With respect to AMPK activity in beta cells, this can also be stimulated by the hormone fibroblast hormone 21 (FGF21) [101]. Recent studies show that FGF21 levels are markedly elevated in people who habitually consume plant-based (vegan) diets of modest protein content—likely reflecting hepatic activation of GCN2, which detects a relative deficiency of essential amino acids [102,103,104]. An epidemiological study in Loma Linda, which has a high population of vegetarians, found that, after multivariate adjustment including BMI, long-term vegans, as opposed to omnivores, were 62% less likely to develop type 2 diabetes, without adjustment for BMI (which tends to be low in vegans), and 77% protection was observed [105]. The low proportion of total dietary fat provided as saturated fat in most vegan diets may also provide protection in this regard by improving peripheral insulin sensitivity and hence lessening glucose/lipid overexposure [106].

## 5. Amplifying or Mimicking the Insulin Signal

With respect to insulin/Akt signaling in beta cells, cGMP and protein kinase G (PKG) have been found to promote Akt activation in beta cells, mimicking the impact of insulin. The mechanistic basis of this effect remains unclear, but it is dependent on intact activity of PI3K [107]. cGMP production in beta cells can be directly stimulated by supraphysiological but clinically feasible concentrations of the B vitamin biotin; this can directly activate the soluble guanylate cyclase [108,109]. This phenomenon likely explains, at least in part, the utility of high-dose biotin for aiding glycemic control in rodent models of diabetes [110,111,112,113,114,115,116]. Increased expression of glucokinase and insulin in the beta-cells of diabetes-prone mice supplemented with high-dose biotin indicates that a protective effect of biotin on beta cell function in diabetic or pre-diabetic rodents [116,117]. High-dose biotin also exerts a trophic effect on beta cell proliferation in newborn mice [118]. Limited clinical data also point to a potential role for high-dose biotin in diabetes management, but the possibility that biotin might reduce risk for diabetes in patients with metabolic syndrome has not been assessed. While high-dose biotin is well tolerated clinically, it has the potential to interfere with certain laboratory tests employing biotinylated reagents, and hence should be discontinued temporarily prior to such tests.

Supplementation with myo-inositol can boost insulin/Akt signaling in circumstances in which the availability of this physiologically essential fact is limiting for the production of phosphatidyl-inositides, which are mediators of such signaling [119] (PI3K-mediated addition of a phosphate to the 3 position of the plasma membrane phosphatidyl-inositol-1,4,5-triphosphate is essential for insulin-stimulated Akt activation). Numerous clinical studies reveal that myo-inositol supplementation (typically 1–2 g twice daily) can aid insulin sensitivity in metabolic syndrome associated with polycystic ovarian syndrome (PCOS) and in gestational diabetes [120,121,122,123,124,125]. Intriguingly, such supplementation has been found to be highly effective for prevention of gestational diabetes in women known to be prone to this syndrome [126] (RR = 0.44, 95% CI (0.32, 0.62), *p* < 0.0001, as determined by meta-analysis of four randomized controlled studies). Although limited clinical evidence suggests that supplemental myo-inositol can aid metabolic control in patients with type 2 diabetes, the possibility that it might decrease risk for new onset diabetes in patients with metabolic syndrome, both by aiding maintenance of beta cell function and by lessening peripheral insulin resistance, has not been tested [127]. The strong utility of myo-inositol for the prevention of gestational diabetes encourages this hypothesis.

## 6. Boosting Glucagon-like Peptide-1 Production for Support of GSIS

With respect to intestinal GLP-1 production, this can be aided by insuring a good population of the colon with “friendly” bacteria that are capable of generating short-chain fatty acids (as by use of effective probiotics), and by the ingestion of prebiotics or of diets rich in soluble fiber and/or resistant starch that deliver fermentable carbohydrate to the distal intestine and colon [128,129,130,131,132]. Curiously, foods high in insoluble fibers (particularly wheat bran) that neither are fermentable nor influence glycemic index are linked to decreased diabetes risk; this may reflect the fact that such foods tend to be rich in ferulate conjugates that are partially cleaved during digestion, rendering the ferulic acid bioavailable [133,134,135,136,137]. Hence, such foods tend to be a source of delayed release ferulic acid, yielding a less episodic increase in plasma ferulic acid than the free compound.

## 7. Summing Up

**Figure 1:**
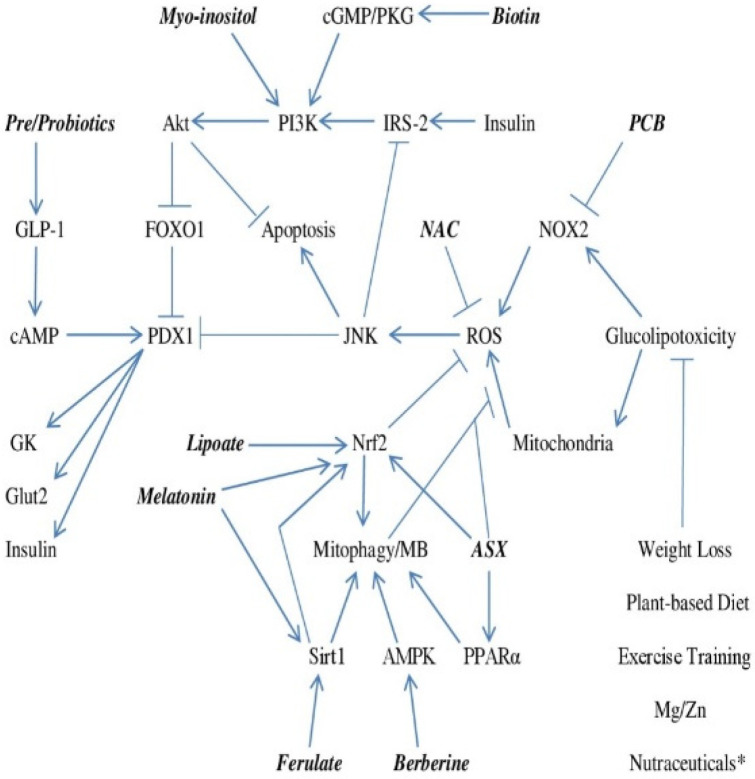
Regulation of GSIS and Apoptosis in Beta Cells Exposed to Glucolipotoxicity–and How Nutraceuticals and Lifestyle May Support Effective Beta Cell Function and Survival. * Nutraceuticals including berberine, myo-inositol, biotin, PCB, cinnamon extract and chromium may diminish beta cell exposure to glucose and free fatty acids by aiding peripheral insulin sensitivity or decreasing hepatic glucose output.

A safe, practical strategy for preventing the conversion of metabolic syndrome to type 2 diabetes could be of tremendous benefit, given the horrendous medical and financial implications of the worldwide rise in diabetes prevalence. This essay has attempted to define—in a vastly simplified form—the molecular biology underlying the glucolipotoxicity-induced failure of beta cell GSIS and the apoptotic loss of beta cells that ushers in overt diabetes, and to postulate nutraceutical strategies with the potential for rectifying the mechanisms driving this beta cell failure. These strategies are intended to do the following: control beta cell ROS production via inhibition of NOX2 and maintenance of healthful mitochondrial function by promoting mitophagy and MB, boost or mimic autocrine insulin signaling, and enhance GLP-1 secretion. Nutraceuticals identified with potential for promoting one or more of these aims include PCB, ferulic acid, lipoic acid, melatonin, berberine, astaxanthin, myo-inositol, high-dose biotin, and pre/probiotics. Figure 1 outlines in schematic form the potential contribution of these nutraceuticals in this regard. Complex supplements and/or functional foods providing a selection of these nutraceuticals may in the future find a practical role in diabetes prevention.

It should be noted that some of the nutraceuticals discussed here may aid diabetes prevention not only through direct effects on beta cells, but also by improving peripheral insulin sensitivity or by aiding postprandial glycemic control, thereby mitigating episodes of glucolipotoxicity. These include berberine, myo-inositol, high-dose biotin, and PCB (from whole spirulina or phycocyanin) [58,66,111,112,113,114,115,125,138,139,140]. Evidently, appropriate weight loss, exercise training, and whole-food plant-based diets also have an important potential in this regard. Ample intakes of magnesium and zinc, and nutraceuticals such as chromium picolinate and cinnamon extract, may also be beneficial [141,142,143,144,145,146,147,148,149].

## Data Availability

No original data.
